# Supplementary material for: Impact of Cellulose-Rich Organic Soil Amendments on Growth Dynamics and Pathogenicity of Rhizoctonia solani
Source: Microorganisms. 2021 Jun 12;9(6):1285. doi: 10.3390/microorganisms9061285 (PMC8231496; doi:10.3390/microorganisms9061285)
Supplement: Supplementary file 1 [file microorganisms-09-01285-s001.zip › microorganisms-1227869-supplementary.pdf]

Supplementary Information belonging to the manuscript entitled *"Impact of cellulose-rich organic soil amendments on growth dynamics and pathogenicity of* Rhizoctonia solani*"*

Anna Clocchiatti, S. Emilia Hannula, Muhammad Syamsu Rizaludin, Maria P. J. Hundscheid, Paulien J. A. klein Gunnewiek, Miriam T. Schilder, Joeke Postma and Wietse de Boer

Table of contents:

Table S1. Performance of *R. solani* on woody substrates and paper pulp.

Table S2. Effect of organic amendments on qPCR-based fungal abundance.

Table S3. Effect of organic amendments on qPCR-based abundance of *R. solani*.

Table S4. Fungal and *R. solani* abundance in soil and roots amended with five organic materials and sown at three time points after amendment.

Table S5. Permutational multivariate analysis of variance (ADONIS) of fungal and bacterial community composition.

Figure S1. Fungal abundance in control soil and soil amended with oak and paper pulp, as based on qPCR and ergosterol measurement (ToS experiment).

Figure S2. Effect of oak sawdust and paper pulp amendment on the fungal community composition in soil and roots.

Figure S3. Effect of oak sawdust and paper pulp amendment on the bacterial community composition in soil and roots.

Figure S4. Effect of oak sawdust and paper pulp amendment on fungal classes.

Figure S5. Effect of oak sawdust and paper pulp amendment on bacterial classes.

Figure S6. Effect of paper pulp as compared to control on bacterial families as found in the roots of beetroot seedlings sown at three time intervals after amendment.

Figure S7. Effect of oak (**A**) and paper pulp amendment (**B**) as compared to control on fungal families in soil sampled 7 days after amendment.

Figure S8. Effect of oak sawdust (**A**) and paper pulp amendment (**B**) as compared to control on bacterial families in the roots of beetroot seedlings.

Table S1. Performance of *R. solani* on woody substrates and paper pulp. Mean and standard deviation (n = 5) for area and local ergosterol concentration of the substrate in the area covered by *R. solani* mycelium. Compact letters show significant differences between substrates for each measurement at $p < 0.05$ (Tukey's test).

**Substrate**	**Area (cm^3)**		**Ergosterol (µg g^{-1})**	
Beech	20.7 ± 0.8	e	19.3 ± 3.6	bc
Oak	13.5 ± 0.7	d	48.8 ± 8.4	de
Hazel	26.0 ± 1.0	ef	42.9 ± 13.8	cde
Black alder	22.7 ± 1.0	ef	41.5 ± 3.5	cde
Birch	21.0 ± 2.6	e	30.3 ± 2.8	bd
Walnut	4.8 ± 1.4	b	26.5 ± 12.4	bd
Maple	12.1 ± 2.0	cd	29.1 ± 1.0	bd
Elder	44.0 ± 3.2	g	14.6 ± 3.6	b
Holly	9.3 ± 1.4	c	55.3 ± 10.0	de
Willow	54.7 ± 1.3	g	38.9 ± 4.4	cde
Hawthorn	13.2 ± 1.4	d	70.0 ± 6.5	e
Snowy mespilus	21.3 ± 2.0	e	53.7 ± 8.6	de
Cypress	0.9 ± 0.1	a	3.3 ± 3.8	a
Douglas fir	3.7 ± 0.3	b	62.1 ± 29.8	de
Paper pulp	31.7 ± 9.7	f	304.8 ± 100.0	f

Table S2. Effect of organic amendments on qPCR-based fungal abundance. Pots were sown at three time intervals after amendment (ToS) and fungal abundance was measured in soil at consecutive days during soil incubation and plant growth (day). Results of ANOVA after generalized linear model with gamma distribution of errors. $R^2 = 0.83$ (Hosmer-Lemeshow). Model $\chi^2 = 350.3$, $p < 0.001$.

	**df**	**deviance**	**Residual df**	**Residual deviance**	**F**	**p**	
Null			509	487.6			
Amendment	5	30.8	502	455.8	35.2	$< 2\ 10^{-16}$	***
ToS	2	1.0	507	486.6	2.9	0.06	.
Day	1	307.3	501	148.5	1,757.2	$< 2\ 10^{-16}$	***
ToS x Amendment	10	3.5	491	144.9	2.0	0.03	*
ToS x Day	2	0.9	489	144.0	2.6	0.07	.
Amendment x Day	5	54.1	484	89.9	61.9	$< 2\ 10^{-16}$	***
ToS x Amendment x Day	10	6.1	474	83.9	3.50	$0{,}2\ 10^{-3}$	***

v

Table S3. Effect of organic amendments on qPCR-based abundance of *R. solani*. Pots were sown at three time intervals after amendment (ToS) and *R. solani* abundance was measured in soil at consecutive days during soil incubation and plant growth (day). Results of ANOVA after generalized linear model with Poisson distribution of errors. $R^2 = 0.85$ (Hosmer-Lemeshow). Model $\chi^2 = 470.3$, $p < 0.001$.

	**df**	**deviance**	**Residual df**	**Residual deviance**	**F**	**p**	
Null			506	553.0			
Amendment	5	95.7	499	307.5	19.1	$< 2\ 10^{-16}$	***
ToS	2	149.8	504	403.2	74.9	$< 2\ 10^{-16}$	***
Day	1	191.3	498	116.1	191.3	$< 2\ 10^{-16}$	***
ToS x Amendment	10	1.6	488	114.5	0.2	1.00	
ToS x Day	2	24.5	486	90.0	12.3	$4{,}8\ 10^{-6}$	***
Amendment x Day	5	6.9	481	83.0	1.4	0.22	
ToS x Amendment x Day	10	0.4	471	82.7	0.04	1.00	

Table S4. Fungal and *R. solani* abundance in soil and roots amended with five organic materials and sown at three time points after amendment. qPCR-based fungal (10^7 copies g^{-1} soil, mean ± *sd*, *n* = 5) and *R. solani* abundance (10^7 copies g^{-1} soil, mean ± sd, *n* = 5) is shown for the control and each treatment in soil sampled during plant germination (one week after sowing), at harvesting (three weeks after sowing) and in root samples. For each column, summary statistics are given for two-way ANOVA. Significant differences are shown for amended soil as compared to the control (Dunnett's test), * 0.05 > *p* > 0.01; ** 0.01 > *p* > 0.001, *** *p* < 0.001.

		Total fungi, soil sampled at germination		Total fungi, soil sampled at harvesting		Total fungi, root samples		*R. solani*, soil sampled at germination		*R. solani*, root samples	
Control	T1	6.3 ± 1.1		14.5 ± 7.03		102.0 ± 28.4		0.0 ± 0.0		2.1 ± 2.5	
	T2	7.5 ± 1.0		26.1 ± 5.9		91.5 ± 36.5		0.0 ± 0.0		0.2 ± 0.2	
	T3	6.8 ± 0.8		19.3 ± 4.9		106.0 ± 25.6		0.0 ± 0.0		0.02 ± 0.01	
Beech	T1	**10.0 ± 2.8**	.	**28.6 ± 10.0**		65.3 ± 10.7		0.0 ± 0.0		0.8 ± 1.2	
	T2	**22.8 ± 2.1**	***	**28.8 ± 7.6**	*	72.8 ± 27.5		0.0 ± 0.0		**0.01 ± 0.01**	*
	T3	**17.6 ± 2.1**	***	**33.3 ± 15.1**		67.7 ± 8.8		0.0 ± 0.0		0.01 ± 0.01	
Oak	T1	**14.3 ± 3.4**	***	**41.7 ± 8.5**		97.8 ± 12.2		0.0 ± 0.0		0.4 ± 0.5	
	T2	**35.2 ± 8.7**	***	**49.0 ± 15.8**	***	134.0 ± 33.5		0.0 ± 0.0		**0.01 ± 0.01**	*
	T3	**37.6 ± 11.9**	***	**41.2 ± 12.1**		78.0 ± 32.5		0.0 ± 0.0		0.01 ± 0.01	
Paper pulp	T1	**11.0 ± 4.4**	*	**10.6 ± 1.9**		101.0 ± 46.7		**0.4 ± 0.4**	**	**9.8 ± 8.7**	*
	T2	**11.5 ± 1.7**	*	**11.1 ± 2.4**	***	57.5 ± 19.7		0.02 ± 0.05		0.06 ± 0.06	
	T3	**11.6 ± 1.3**	**	**15.9 ± 4.4**		80.0 ± 8.2		0.1 ± 0.2		0.01 ± 0.01	
Hair meal	T1	6.6 ± 1.6		**10.8 ± 5.8**		**42.6 ± 16.8**	**	0.0 ± 0,0		0.6 ± 0.6	
	T2	10.1 ± 3.8		**12.7 ± 3.1**	***	77.1 ± 29.9		0.0 ± 0.0		**0.05 ± 0.1**	.
	T3	8.5 ± 2.5		**14.2 ± 6.0**		92.7 ± 49.0		0.0 ± 0.0		0.05 ± 0.1	
Shrimp meal	T1	6.7 ± 1.9		**10.1 ± 2.4**		**50.5 ± 14.3**	*	0.0 ± 0.0		0.3 ± 0.2	
	T2	8.2 ± 0.9		**10.3 ± 1.0**	***	62.6 ± 15.5		0.0 ± 0.0		**0.02 ± 0.03**	*
	T3	7.9 ± 1.0		**10.3 ± 3.8**		**56.4 ± 20.0**	*	0.0 ± 0.0		0.0 ± 0.0	
Treatment		$F_{5.90} = 72.4$ ***		$F_{5.90} = 46.8$ ***		$F_{5.90} = 7.0$ ***		$F_{5.90} = 6.8$ ***		$F_{5.90} = 7.6$ ***	
ToS		$F_{2.90} = 29.1$ ***		$F_{2.90} = 0.02$ *		$F_{2.90} = 0.4$		$F_{2.90} = 3.2$ *		$F_{2.90} = 38.7$ ***	
TreatmentxToS		$F_{10,90} = 4.0$ ***		$F_{10,90} = 1.2$		$F_{10,90} = 2.7$ **		$F_{10,90} = 3.0$ **		$F_{10,90} = 7.3$ ***	

Table S5. Permutational multivariate analysis of variance (ADONIS) of (**A**) fungal and (**B**) bacterial community composition, using Bray-Curtis dissimilarity matrix partitioned by soil amendment (control, oak sawdust, paper pulp), sowing time after amendment (ToS) and sampling day. A blocking variable was introduced as a constraint to the permutations (strata). The relative abundance data were log + 1 transformed before the analysis. SS: sum of squares, MS: mean sum squares. a: significance values based on 9999 permutations.

**A**	***df***	***SS***	***MS***	**pseudo-*F***	R^2	p^a	
Amendment	2	14.2	7.1	76.5	0.3	0.0001	***
ToS	2	0.5	0.2	2.2	0.01	0.018	*
Day	5	15.3	3.1	33.0	0.3	0.0001	***
ToS x Amendment	4	0.7	0.2	1.85	0.02	0.019	*
Residuals	181	16.8	0.1		0.4		
Total	194	47.4			1		
B	***df***	***SS***	***MS***	**pseudo-*F***	R^2	p^a	
Amendment	2	1.9	0.9	2.3	0.02	0.0001	***
ToS	2	0.9	0.5	11	0.01	0.12	
Day	5	6.7	1.4	3.3	0.08	0.0001	***
ToS x Amendment	4	1.8	0.4	1.1	0.02	0.14	
Residuals	181	75.4	0.4		0.9		
Total	194	86.7			1		

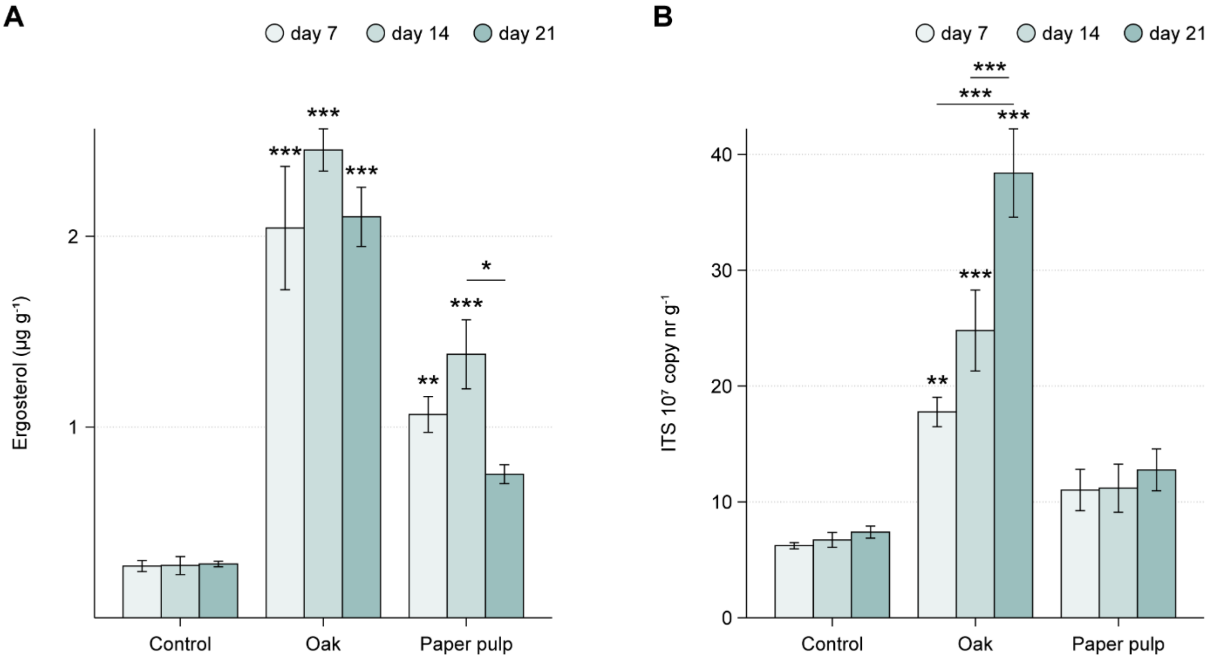

Figure S1. Fungal abundance in control soil and soil amended with oak and paper pulp, as based on qPCR and ergosterol measurement (ToS experiment). Fungal abundance is shown for pots of the T3 series, sampled on day 7, 14 and 21. Significant differences between each treatment and the control (Dunnett's test) are indicated on the top of each bar as * 0.05 > p > 0.01; ** 0.01 > p > 0.001, *** p < 0.001. Whereas comparisons among days within each treatment are shown above horizontal lines (Dunnett's test).

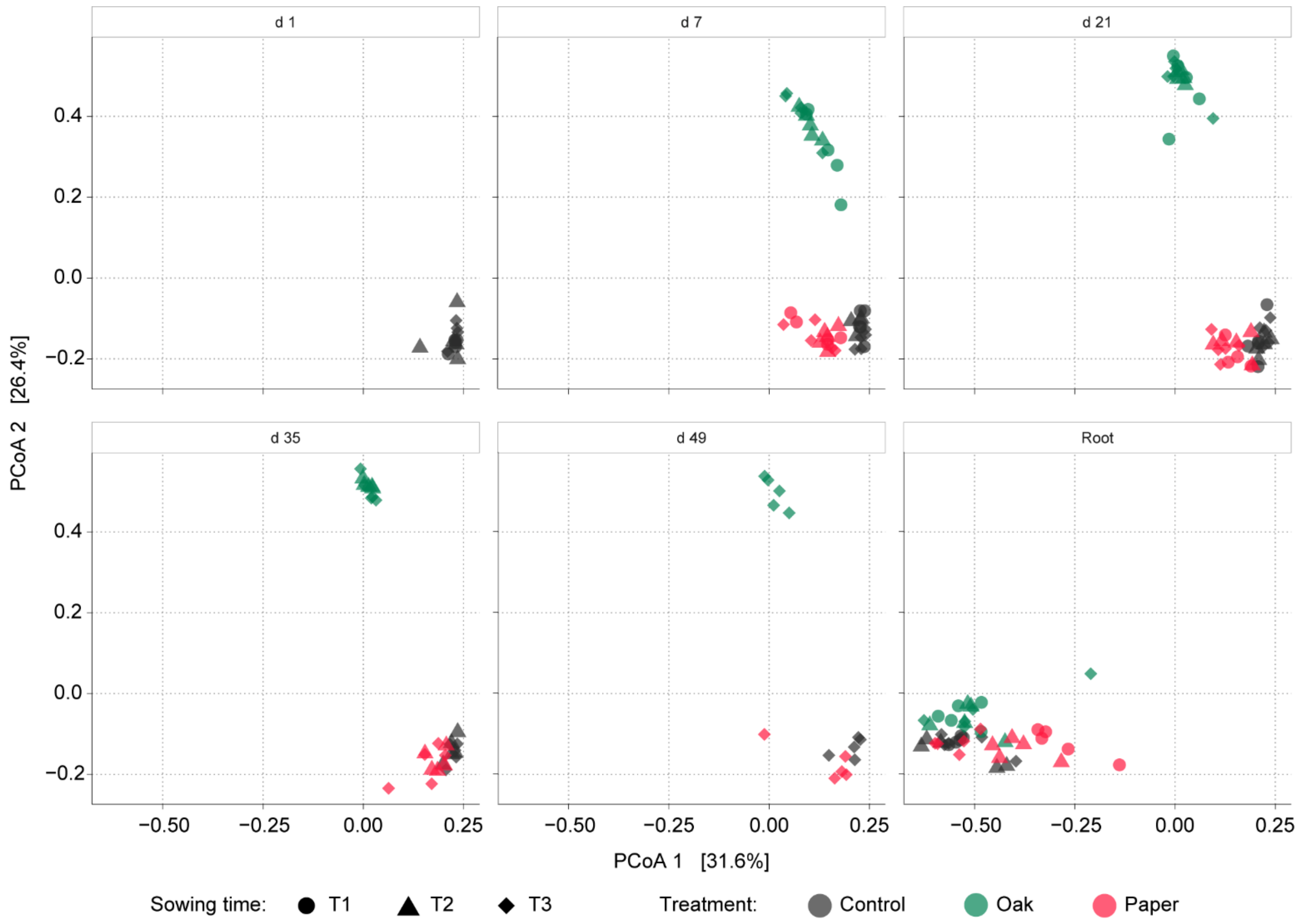

Figure S2. Effect of oak sawdust and paper pulp amendment on the fungal community composition in soil and roots, in pots at three time points of sowing and harvesting after amendment (T1-3; see Fig. 2 main article). The results of the same PCoA ordination analysis (based on a Bray-Curtis distances) are plotted separately for soil sampled at day 7, 21, 35, 49 and for root samples.

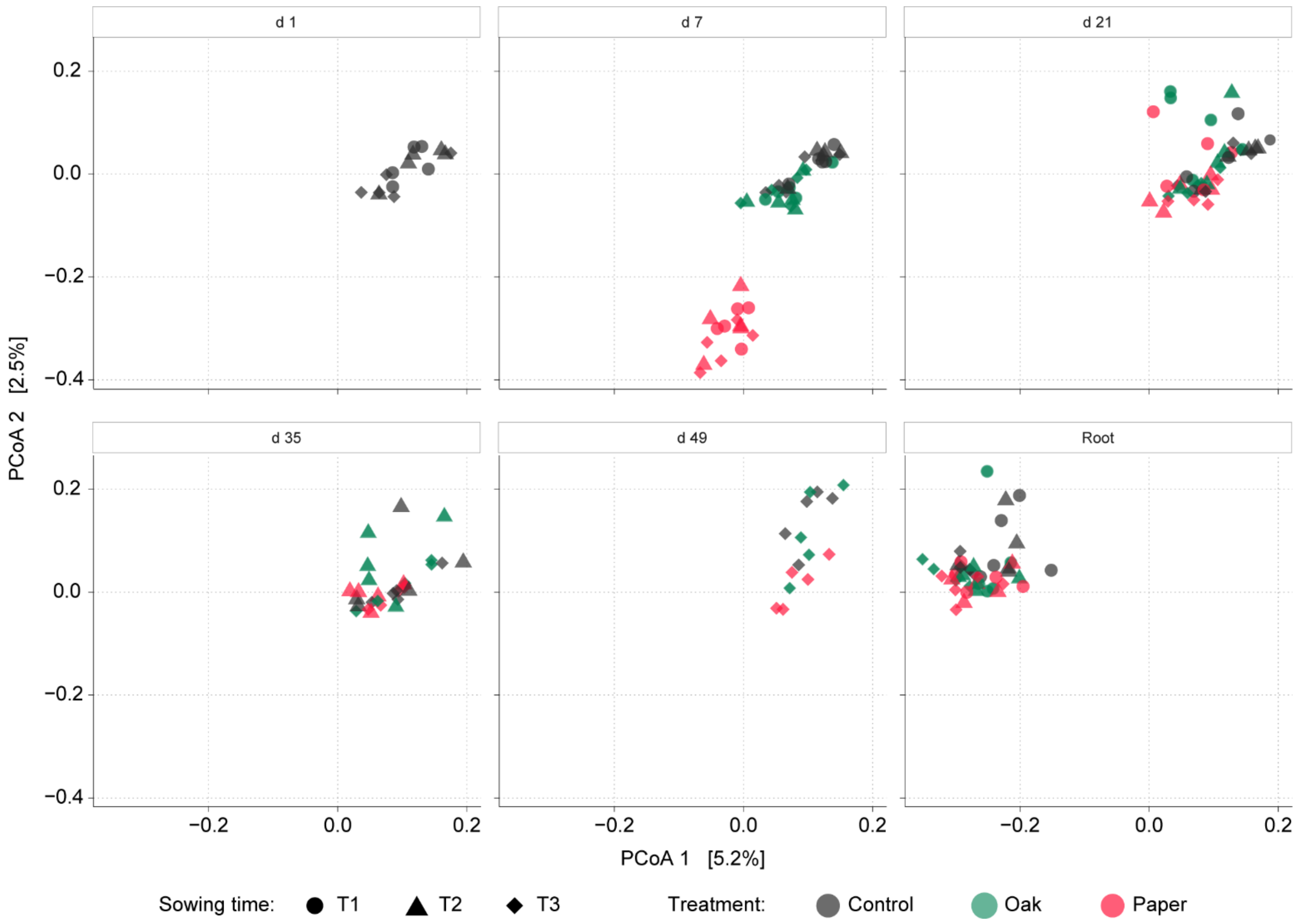

Figure S3. Effect of oak sawdust and paper pulp amendment on the bacterial community composition in soil and roots, in pots at three time points of sowing and harvesting after amendment (T1-3; see Fig. 2 main article). The results of the same PCoA ordination analysis (based on a Bray-Curtis distances matrix) are plotted separately for soil sampled at day 7, 21, 35, 49 and for root samples.

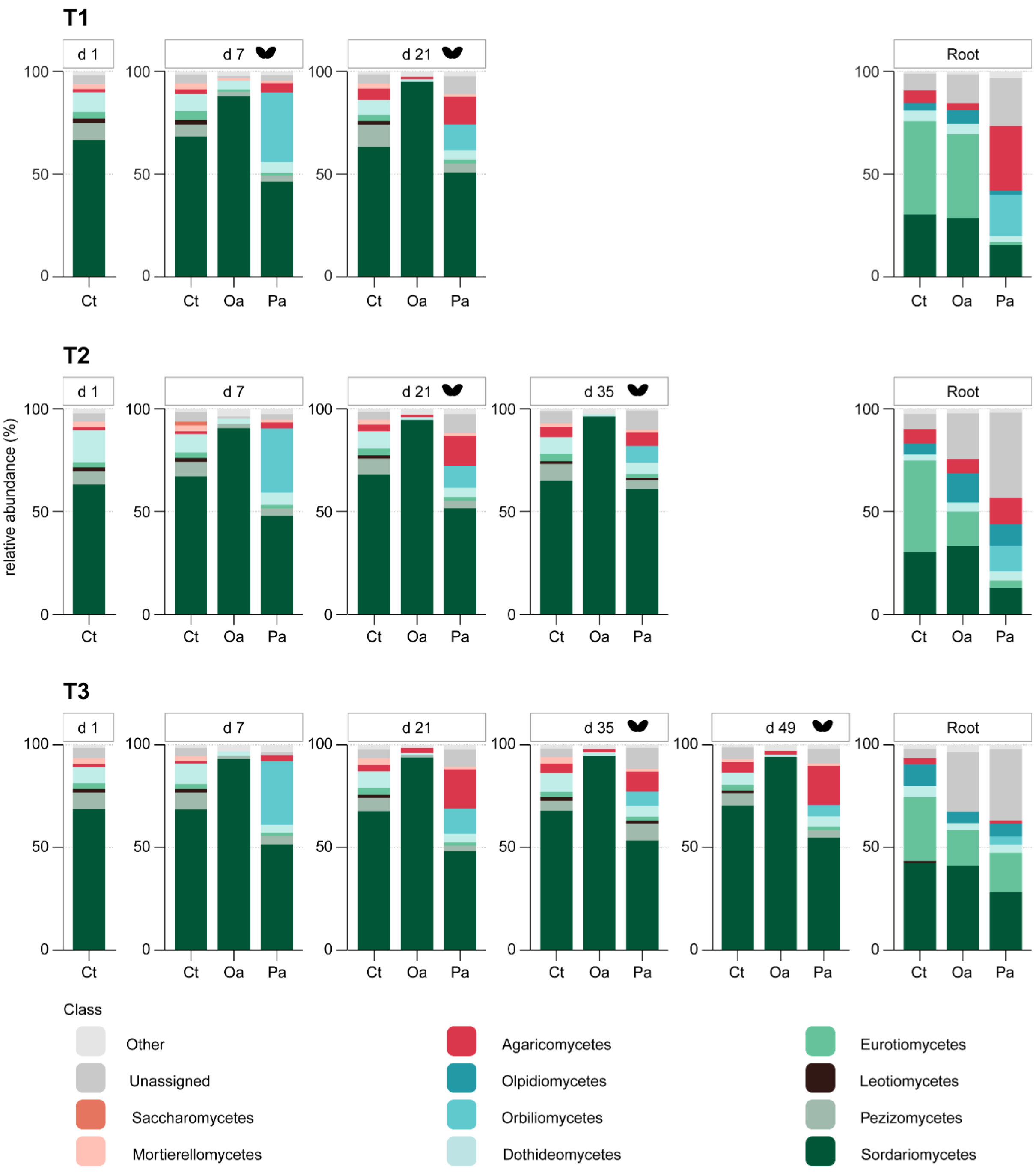

Figure S4. Effect of oak sawdust and paper pulp amendment on fungal classes. Relative abundance of fungal classes are displayed for soil collected at 7, 21, 35 and 49 days after amendment and for the root interior. Data are shown for both unplanted and planted pots (marked with a plant symbol). Pots were sown at three time points after amendment (T1, T2 and T3; see Fig. 2 main article). Fungal classes with proportion < 1.5% are classified as "Other".

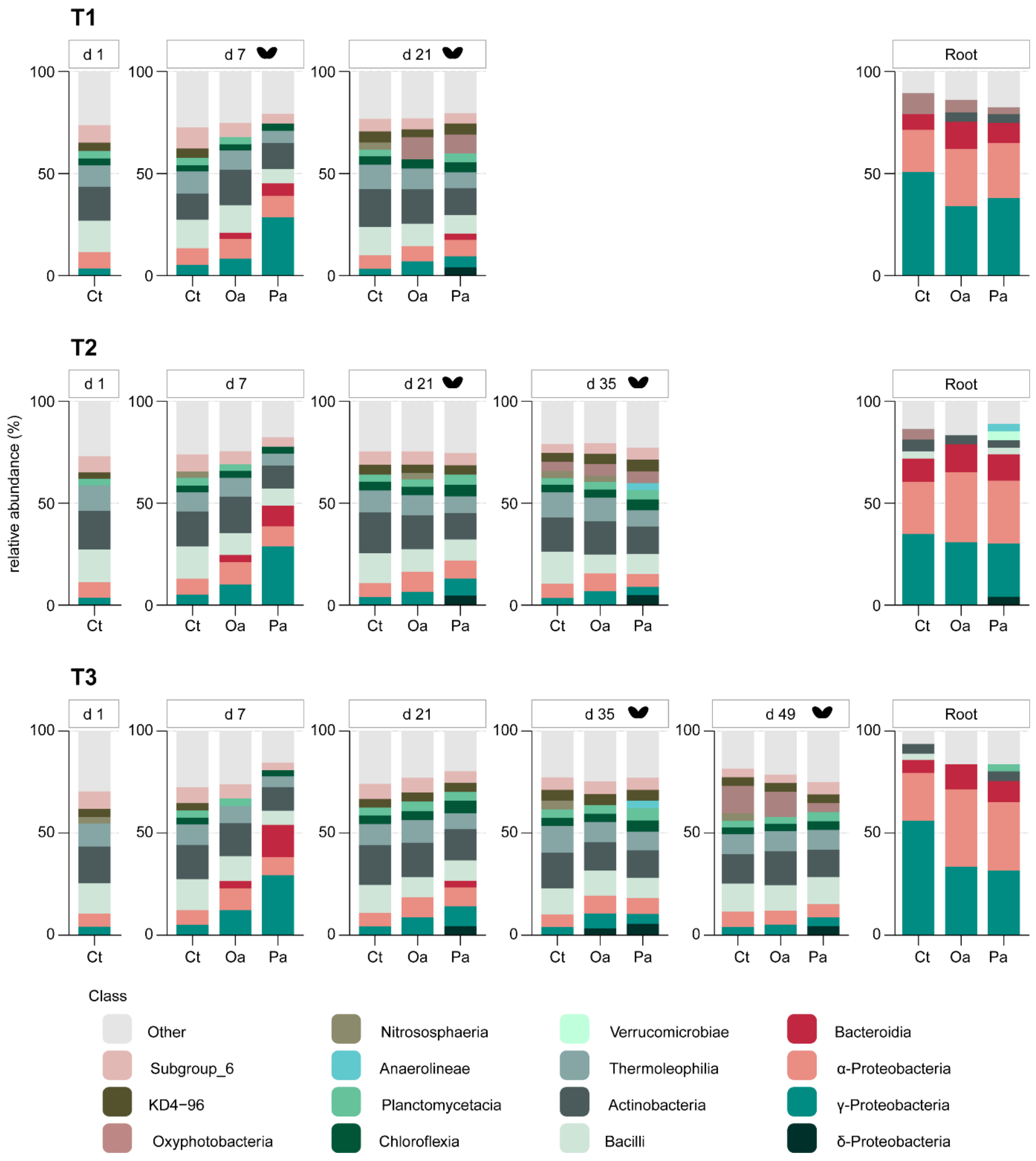

Figure S5. Effect of oak sawdust and paper pulp amendment on bacterial classes. Relative abundance of bacterial classes are displayed for soil collected at 7 , 21, 35 and 49 days after amendment and for the root interior. Data were obtained for both unplanted and planted pots (marked with a plant symbol). Pots were sown at three time points after amendment (T1, T2 and T3; see Fig. 2 main article). Bacterial classes with proportion < 3% are classified as "Other".

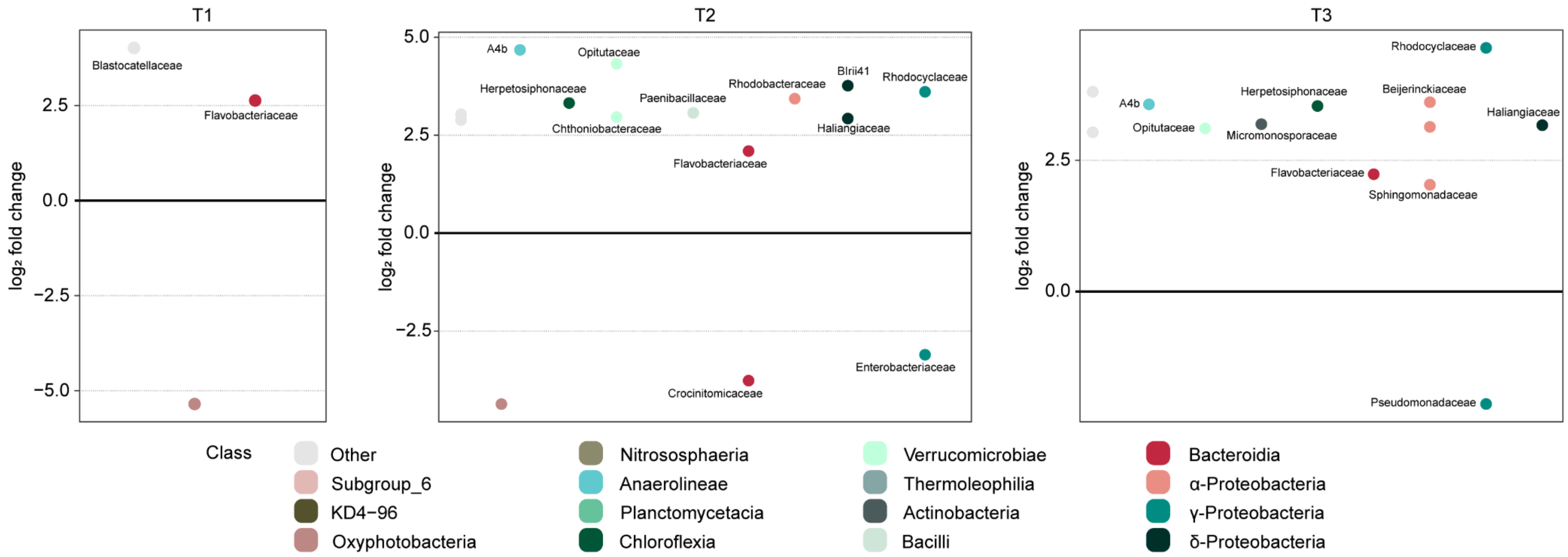

Figure S6. Effect of paper pulp as compared to control on bacterial families as found in the roots of beetroot seedlings sown at three time intervals after amendment. Bacterial families significantly over- ($\log_2$ fold change > 0) and under-represented ($\log_2$ fold change < 0) were highlighted by differential abundance analysis (Wald test $p < 0.05$), performed for T1, T2 and T3. Bacterial families are grouped by class along the x-axis. Bacteria belonging to classes with relative abundance < 1.5% are indicated as "Other".

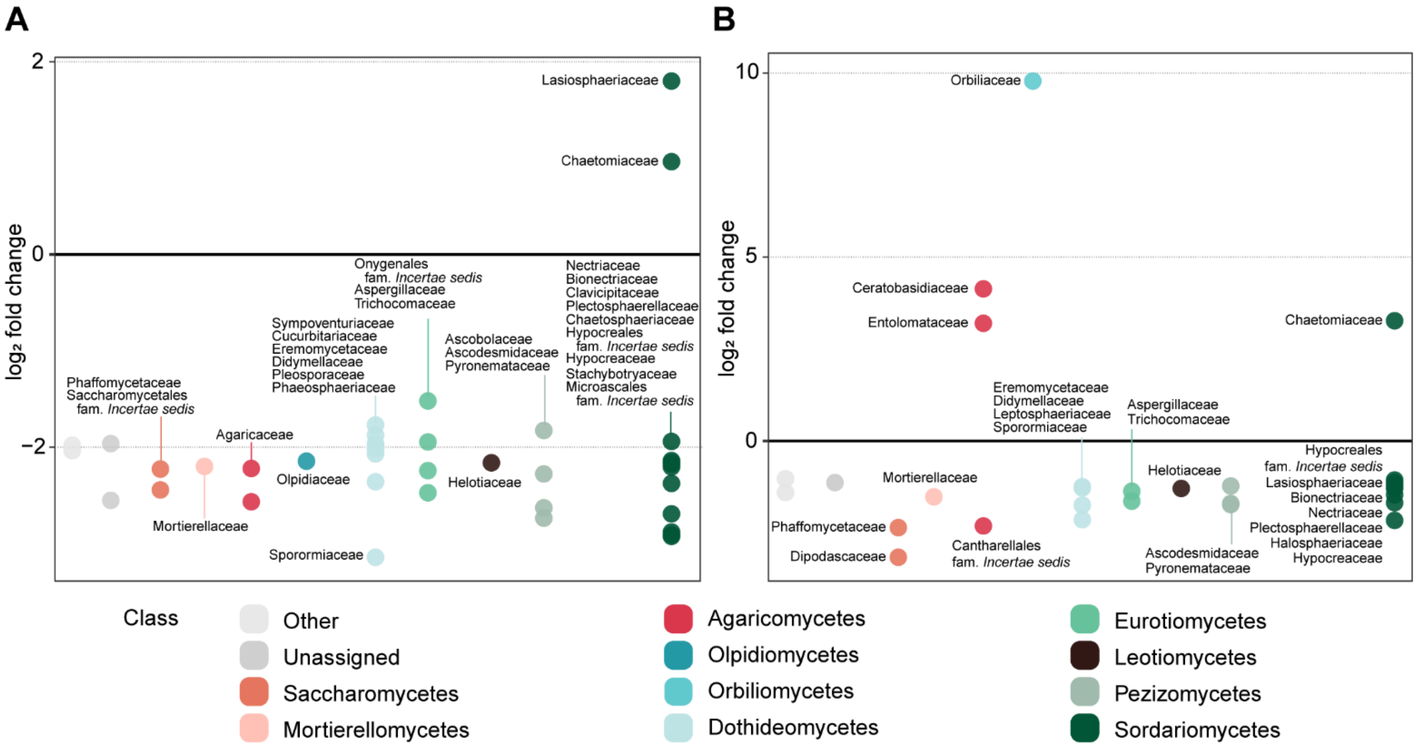

Figure S7. Effect of oak (**A**) and paper pulp amendment (**B**) as compared to control on fungal families in soil sampled 7 days after amendment. Fungal families significantly over- (log_2 fold change > 0) and under-represented (log_2 fold change < 0) were highlighted by differential abundance analysis (Wald test $p < 0.01$). Fungal families are grouped by class along the x-axis and fungi belonging to classes with relative abundance < 1.5% are indicated as "Other".

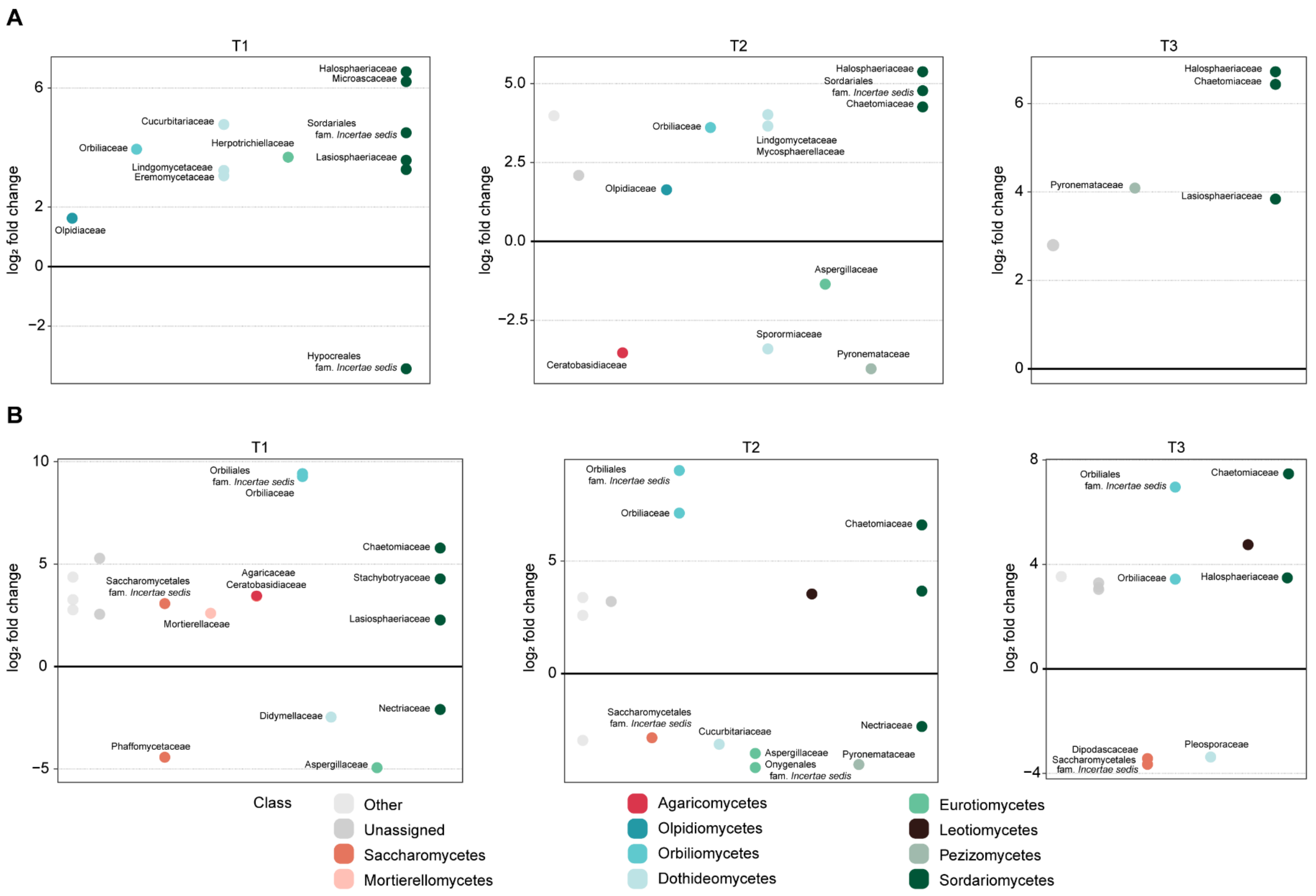

Figure S8. Effect of oak sawdust (**A**) and paper pulp amendment (**B**) as compared to control on bacterial families in the roots of beetroot seedlings. Bacterial families significantly over- ($\log_2$ fold change > 0) and under-represented ($\log_2$ fold change < 0) were highlighted by differential abundance analysis (Wald test $p < 0.05$) for T1, T2 and T3. Bacterial families are grouped by class along the x-axis. Classes with relative abundance < 3% are indicated as "Other".
